# Online Groups and Patient Forums

**DOI:** 10.1007/s11920-014-0507-3

**Published:** 2014-10-02

**Authors:** Sabina Dosani, Claire Harding, Simon Wilson

**Affiliations:** 1 Big White Wall Ltd, 104-110 Goswell Road, London, EC1V 7DH UK; 2 Anna Freud Centre, 12 Maresfield Gardens, London, NW3 5SU UK; 3 Institute of Psychiatry, King’s College London, 16 De Crespigny Park, London, SE5 8AF UK

**Keywords:** Social media, Online social networking tools, Online self help forums, Online communication, Bipolar disorders, Depression, Online therapy, Suicide, Self-harm, Pro-anorexia

## Abstract

Online mental health support forums are becoming increasingly popular and there is evidence that they are useful: particularly for providing anonymous support and filling information gaps. However, there are also very real concerns about negative outcomes for users. One online mental health service, Big White Wall, manages these risks and supports its members through the provision of 24 hour professional moderation. Comparison of Big White Wall’s member population with the population of one London borough shows a diverse user group, but members are more likely to be female, and aged 25 to 34, or unemployed.

## Introduction

Many of us live part of our lives in a digital universe. Online support groups and communities have radically changed the ways in which we communicate and connect, and being part of an online community is an important part of many people’s lives. It is easy to see why: physical co-location no longer matters. The ability to be online, rather than an accident of geography, now means people with similar life-circumstances or predicaments, often miles apart, are connecting and establishing supportive relationships. The past decade has seen countless online self-help forums forming and many joining: not only to discuss their problems, but also to offer their peers advice and support. Many of those living with mental distress, others who may be struggling with life’s vicissitudes, and those with diagnosed mental illnesses have found these forums beneficial. The advantages of health literacy are well known. However, the potential pitfalls of online groups possibly worsening existing conditions, by encouraging harmful behaviour including self-starvation, self-harm and suicide have had considerable media attention. In this paper, we evaluate the literature published over the past three years in this field, report on observations we have made at the online mental health service, Big White Wall, and offer our view of how online support forums are changing the face of mental health care.

## What is an Online Group?

Online groups are digital communities affording members opportunities to interact with one another using the internet. Many online communities, including most online support groups, are also repositories of helpful information and advice. Members usually have to ‘sign up’ to the community and agree to abide by certain rules and expected behaviours. There are many ways for members to interact in online groups, including taking part in diagnosis-specific patient forums, in chat rooms or by subscribing to email lists.

## Characteristics of Online Support Groups (for Side Box)

- Virtuality
- Shared goals
- Media richness
- Support network
- Lack of physical co-location
- Computer connects members rather than geography
- People report they feel more able to talk about hidden conditions eg being transexual
- Relief of telling a personal story to strangers and not feeling alone

## Group Behaviour Online: A Review of the Recent Literature

In 2011, Ray Jones, Siobhan Sharkley and colleagues, in Plymouth, UK, explored what young people who self-harm think about online self-harm discussion forums. ‘SharpTalk’, an experimental user discussion forum, was set up to facilitate shared learning between health professionals and young people who self-harm [1]. The Plymouth team extracted themes and illustrative statements from online discussions and asked participants to rate statements. Respondents said they learned more about mental health issues from online discussion forums than from information sites, found it easier to talk about self-harm to strangers than to family or friends, and preferred to talk online than face-to-face or on the telephone. They valued the anonymity the forums provided and reported feeling more able to disclose and less likely to be judged online than in ‘real life’. The authors concluded that mental health professionals should be aware of the value of anonymous online discussion forums for some young people who self-harm, so that they can talk about them and assess their use with their patients.

Similar findings regarding the value of anonymity were made in an Australian study, with service development conducted some years prior [2]. In 2004 a free-to-the public, online peer-to-peer bulletin board BlueBoard (blueboard.anu.edu.au) was developed and embedded in the Australian depression information website, BluePages (bluepages.anu.edu.au). This board was established and hosted by the Depression and Anxiety Consumer Research Unit at The Australian National University. It was established to offer support to people suffering from depression, and for their friends and caregivers, with the aim of enabling anonymous interaction and mutual support. Posting material referring to suicidal thoughts or self-harm was strictly prohibited.

A qualitative investigation concluded that the anonymity afforded by these discussion forums enabled people to open up to a greater degree than they would have otherwise. Many people self-reported benefits from these forums. Questions remained, however over the adequacy of psycho-educational materials. Many users were left with questions, including, is adequate and appropriate information, related to recognising and understanding depression, available through these forums? Is adequate and appropriate information available about medicines and treatment?

Colleagues from the USA commented that adults routinely use the internet as a source of health information [3•] and the Oxford Internet Survey 2013 found that 69 % of current internet users used the internet for health information in the UK [4]. Monteith et al. recommended that patients with bipolar disorder and their caregivers should be encouraged to increase their knowledge of this complex illness, including accessing information online [3•]. They cautioned though, that patients, caregivers, and physicians should be aware of potential perils when searching for health information, such as loss of privacy, poor quality content, and internet scams. They ended by recommending that physicians should provide patients with a list of trustworthy websites.

People living with bipolar disorder have also reportedly found online forums helpful, citing friendship, empathy and online group cohesion [5]. Users report finding online self-help forums optimal for discussing their problems because of the anonymity involved. In many online groups, people use pseudonyms and this can reduce the risk that they may share harmful content that may lead to harm to others.

## Example of a Digital Service — Observations at Big White Wall

Founded in 2007, Big White Wall is an online mental health service for over 16 s, currently available in the UK, the USA and New Zealand. Members access the service by self-referral (by subscribing privately, via their local health service or employer), or by being referred by various health professionals, mostly in primary care. The service is also available to serving and former UK military personnel and their families. It aims to promote independence, and members are discouraged from remaining with the online community for more than six months.

Our user Support Network, a vibrant, online community, is the heart of the service. An anonymous peer-support system, designed for those with mild to moderate mental health problems. The model is one of user empowerment and self-support.

**Fig. 1:**
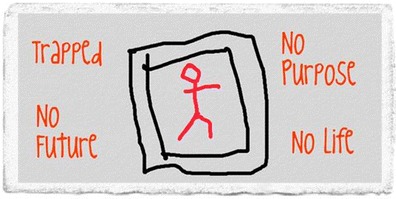
Brick reads ‘trapped, no purpose, no future, no life’. Line drawing of a person inside two rectangular boxes. Figure was created by BWW members and are shared with permission

**Fig. 2:**
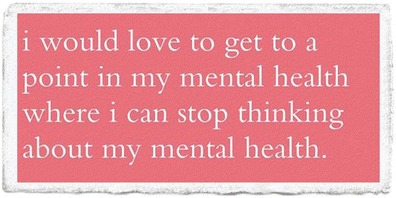
Brick reads ‘i would love to get to a point in my mental health where i can stop thinking about my mental health’. Figure was created by BWW members and are shared with permission

**Fig. 3:**
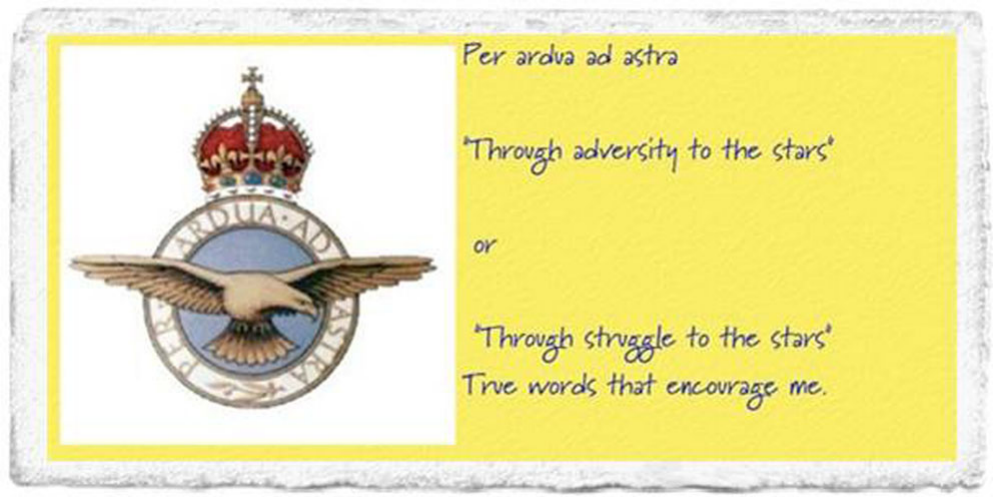
Brick reads ‘per ardua ad astra “through adversity to the stars” or “through struggle to the stars” True words that encourage me.’ Image of Royal Air Force crest. Figure was created by BWW members and are shared with permission

Big White Wall’s members create bricks for the wall: literally hundreds of images every month which show what’s on their mind that day. These can be posted onto the Wall or sent to other members as a gift. Some bricks have swirling grey clouds, some sunshine on a beach, some pictures of a favourite pet. Some are expressions of deep pain and fear (Fig. 1, see also Fig. 2), others celebrations of a small step forward (Fig. 3). One recent brick simply listed all the mental health diagnoses the member had been given; they filled the whole image. Big White Wall allows even those without a formal diagnosis to access support if they feel like they are struggling. As an emotional wellbeing and mental health service, the community can support people with a wide range of needs.

None of this happens in an isolated backwater of cyberspace. Moderators — known as Wall Guides — provide empathic responses if the community hasn’t already done so. They roll out the virtual ‘welcome mat’, enabling new members to feel at home and safe. Working shifts that cover 24 hours, Wall Guides also keep a trained eye out for material that could trigger self-harming impulses in others and take these images down.

Members communicate with one another via a community forum (Community Talkabouts) or one-to-one (Personal Talkabouts). There is also a range of useful support information available on the site in relation to common mental health problems (Useful Stuff), and members can take tests to measure their symptoms more accurately (such as the PHQ-9 questionnaire for depression).

An additional feature of Big White Wall is Guided Support. These programmes are a series of online group interventions for depression, anxiety, and similar disorders, based on CBT and problem-focused therapy. Members sign up to join one of these courses, run by therapists, and new material and homework is posted online each week as the course runs for the group to work on together.

An optional adjunct to these supports is Live Therapy where a member can choose a therapist for sessions of brief focused interventions (CBT or counselling) in real time. This can be text-based, audio, or visual, and the BWW has its own in-house secure video conferencing platform for this purpose.

Wall Guides have qualifications in counselling, social work, psychology or related disciplines. All are trained by BWW in how to manage the SupportNetwork, and a Wall Guide handbook is used to ensure consistency. Live Therapists, who currently work only for UK contracts, are all members of accredited professional bodies for the type of therapy they deliver and have at least three years of post-qualification experience. The Wall Guides and Live Therapists have weekly supervision that is led by consultant psychiatrists and issues are also discussed with a senior Wall Guide on each shift, or with a wider team, including psychiatrists, by email as they arise.

## Managing Potential Harm at Big White Wall

Like all interventions, online groups and patient forums have the ability to cause harm. Members can be hurt by other members, who, while not wishing to cause distress, may not have nuanced their response as carefully as a therapist would.

### Triggering

A ‘trigger’ is something that stimulates a traumatic memory or self-harming behaviour in someone who is psychologically vulnerable. We do not allow images of suicidal behaviour or self-harm on the Wall, in an endeavour to protect members from triggering. However, we also recognise that this is not an exact science and discussions about which images may or may not be potentially triggering cause lively debates both within our team and among our members, who are understandably disappointed if their brick is ‘censored’. We welcome research into this area of potentially triggering images to ensure our decision making is fair and evidence-based.

### Trolling and Flaming

Those who post inflammatory comments, insults, abuse or hateful remarks online are known as ‘trolls’. Flaming is the name given to horrible or contemptuous arguments (flames) posted on online discussion forums. There have been some high profile convictions in the UK for those trolling on Twitter and Facebook. At Big White Wall, we do have problems with trolls from time to time. However, round the clock moderation from Wall Guides and removal of membership from those caught trolling helps to reduce the risks of harm to members.

### Managing Crises

Like all mental health services, BWW members sometimes present in crisis. This can be identified from a high score on the suicidal ideation question on the PHQ9, if a member posts a message relating to suicidal feelings or urges to self-harm, or if a member messages using the ‘Ask a Wall Guide’ function. In all cases, the goal is to support the member in the anonymous context of the service, and if necessary to protect other members, and risk is managed according to agreed service protocol. Members will be brought into a one to one text discussion with the Wall Guide on duty, who will assess risk and, if it is high, advise the member to access health services immediately. The Wall Guide will continue to message the member who is in crisis until they know that they are safe: comprehensive handover notes and a member flagging system mean that vulnerable members are tracked consistently between shifts.

## Example Application: Big White Wall and the London Borough of Wandsworth

Big White Wall has been available to residents in the UK’s London Borough of Wandsworth since 2011, commissioned by the National Health Service (NHS) Clinical Commissioning Group (CCG). Between 1 April 2014 and 31 March 2014 686 people joined. A slight majority (383) were referred by their general practitioner (GP), with the remainder joining from the Improving Access to Psychological Therapies (IAPT) service, other health professionals, public health campaigns, website and social media information, and recommendations from others. Entitlement is verified by the use of a Wandsworth postcode, or members can be referred to the service directly by a health professional.

This section compares the demographic profile of members joining Big White Wall from Wandsworth in this period to the population of the borough defined in the 2011 Census [6, 7].

Women were overrepresented among BWW members in Wandsworth (Table 1). Women are similarly over-represented in IAPT delivered primary health care: in 2012/13 Q4, 83 % more women were treated than men [8].

**Table 1:** Gender of BWW members in Wandsworth compared to general population

	Big White Wall	2011 census
Male	26.5 %	48.4 %
Female	73.3 %	51.6 %
Other	0.1 %	NA

Table 2 shows that White British people are over-represented among Big White Wall users in Wandsworth compared to the population overall, with Asian people notably underrepresented. However, the Census figures refer to the population as a whole, and Big White Wall figures only to over 16 s. Big White Wall is also only available in English, and 2.4 % of Wandsworth residents speak English ‘not well’ or ‘not at all’.

**Table 2:** Ethnicity of BWW members in Wandsworth compared to general population

	Big White Wall	2011 census
White British	60.6 %	53.3 %
Other White	19.1 %	18.1 %
Black	7.7 %	10.7 %
Asian	5.0 %	10.9 %
Mixed background	5.1 %	5.0 %
Other background	2.5 %	2.1 %

Big White Wall members are significantly more likely to be aged 25 to 34 than the population as a whole, and significantly less likely to be aged over 55 (Table 3). Most of Big White Wall’s services show a pattern of this type, which may well be related to the epidemiology of depression and anxiety [9]. However, the over-representation of 25-34 year olds is unusually acute in Wandsworth. The borough has very high rates of GP referral, and the age distribution may be related to the assumptions made by GPs about the types of patients using online services: Big White Wall data for 2013 shows that the average age of members referred by GPs is 35.2 years, compared to 38.5 years for all other referral routes [7].

**Table 3:** Age of BWW members in Wandsworth compared to general population

	Big White Wall	2011 census (16+ residents only)
16-24	15.9 %	13.3 %
25-34	47.2 %	34.9 %
35-44	21.6 %	20.4 %
45-54	10.9 %	12.3 %
55-64	3.5 %	8.6 %
65+	0.9 %	10.5 %

Overall 64.4 % of Big White Wall members in Wandsworth are in paid employment, compared to 70.8 % of the population as a whole (Table 4). There is a significantly higher representation of unemployed people using Big White Wall than in the general population. This is likely to be related to the association between unemployment and poor mental wellbeing. Retired people are significantly underrepresented.

**Table 4:** Economic activity of BWW members in Wandsworth compared to general population

	Big White Wall	2011 census (residents aged 16 to 74 only)
Full time	54.5 %	59.4 %
Part time	9.9 %	11.4 %
Student	12.2 %	9.3 %
Unemployed	10.2 %	3.8 %
Looking after home or family	4.8 %	4.3 %
Retired	1.0 %	6.2 %
Long term sick or disabled	2.9 %	2.9 %
Other economically inactive	NA	2.7 %
Other employment status	4.4 %	NA

## Member Stories: Examples from Big White Wall

[Source: transcripts of videos filmed April 2014, shared anonymously with permission from subjects]

### A Member from the Military Community

My son joined the Royal Marines, that's really what happened. He had been out in Afghanistan. They were doing a routine patrol one morning, in doing so this IED — improvised explosive device — blew up catastrophically, and [he] was very seriously injured.

I became his carer, essentially. So I was redressing his wounds and just nasty stuff like that really. And you know, you hear things and you think is he crying, is he OK, what do I do? Who would I ask for support?

People talk about this deep dark space, this deep hole, and it felt like that, I could feel myself going further and further down. I was so shocked at myself, even thinking that I was just low. I realised that I couldn't carry on like this, I had to seek help.

I'd been to a conference, because we were invited as families of the injured, and the Big White Wall was mentioned. And that was the best thing ever. The Big White Wall was absolutely brilliant. It was there night or day. I could write, I could design bricks with artwork, and immediately got responses back.

But each time I had a down moment, which would last usually about two to three weeks, each time, somebody came back. People listened, people understood. I think if you talk about what you're going through it makes such a difference, but if you've got nobody to talk to, what do you do? You know, that's why the Big White Wall was so important — it stopped me slipping down.

The Big White Wall was there when things were really bad. I will forever be grateful for that because it was massive for me, it was a lifesaving device actually.

### A Member who is a Student

I was put on medication for depression when I was 17, and then it really didn't start getting bad until I started university, and it was when I didn't get on with my flatmates that things really kicked off. And that's when I was introduced to Big White Wall.

It's just really nice to talk to people who don't know you, cause then they don't know your past and they can't judge you, so you can talk to someone without being scared that anything will get out of Big White Wall. Like, what happens in Big White Wall stays in Big White Wall. A lot of the time when I used it — you don't really feel like talking to someone, but you can type and share pictures. Even then people would still comment back: 'hope you're OK, what's the matter'.

The comforting thing about it is knowing that there's professionals constantly monitoring it.

I definitely think using Big White Wall gave me more confidence about talking about my issues. After using Big White Wall I started to talk to my friends, people at uni, and then stepped forward from that — people I worked with. And I feel like I've met so many more people through Big White Wall giving me the confidence to talk to others.

I came off anti-depressants about, maybe, 6 months after using Big White Wall. The doctor wanted me to carry on, but I was like 'no, honestly, I found something else'.

I don't think I'd be in the place I am now, definitely. I think I'd still be relying on medication, and still be very against going to counselling. The worst thing for me is people try and force me to do it, whereas Big White Wall they said, you know, like 'I'll give you a username and password', and obviously it's up to you when you want to start using it, if you want to at all, and obviously I chose to, and it's the best decision I've ever made really.

## Conclusion

Recovering from mental illness often requires much more than a medical diagnosis, formulation and medication. Psychoeducation, support from others who have had similar experiences, professional advice and access to therapy that is not bound by traditional ‘office hours’ can all help those struggling with mental ill health to look after themselves better and recover. Advances in technology mean that users and potential users of psychiatric services are using online support networks and guided support groups to cope, adapt and change their mental health for the better.
